# Social interaction and conceptual change pave the way away from children’s misconceptions about the Earth

**DOI:** 10.1038/s41539-019-0051-3

**Published:** 2019-08-28

**Authors:** Diego Pablo de la Hera, Mariano Sigman, Cecilia Ines Calero

**Affiliations:** 1 Laboratorio de Neurociencia, Universidad Torcuato Di Tella, Buenos Aires, Argentina; 2 Consejo Nacional de Investigaciones Científicas y Técnicas (CONICET), Buenos Aires, Argentina

**Keywords:** Human behaviour, Cognitive neuroscience

## Abstract

Throughout development, children undergo moments of abrupt conceptual transitions, often replacing intuitive knowledge with grounded scientific theories. This typically also creates a situation of social conflict, as different children may hold at the same time substantially different theories and explanations about the same phenomenon. The main objective of this work is to understand whether social interaction and exchange of arguments and reasoning may be a catalyzer for conceptual development. Dyads of 7-year-old children with different conceptual understanding of the Earth were asked to reach a consensus about its astronomic and geometric properties. Our results show that mere minutes of deliberation can result in substantial changes in children’s conceptual representations, and moreover, that this transition was consistently in the direction of reasoned and scientific opinions. These results provide empirical evidence and suggest specific ways in which peer interaction can be used effectively to promote conceptual change in school settings, in a knowledge domain at the center of this era’s post truth and science denial crisis.

## Introduction

People often have different and contrasting knowledge and opinions. Science is no exception, and a variety of theories and interpretations are usually available for one same matter. How groups solve conflict and disagreement that result from these differences depends on the nature of the situation, and may involve a variety of strategies leading to different outcomes in collective construction of knowledge. Some such strategies include (a) aggregation of knowledge (i.e., voting) and selection of an unweighted combination or average of all choices,^1,2^ (b) reaching consensus through free interaction among each other, identifying experts, leaders, or sharing confidence estimations,^3–5^ and (c) exchanging arguments,^6^ among others.

However, not only does conflict contribute to collective construction of knowledge, but it has been known for decades to promote individual cognitive development as well.^7,8^ The matter of where knowledge comes from has been addressed by both Piagetian cognitivism and Vygotskian socioculturalism, and their derivatives.^7^ Although both share a constructivist view, with subjects actively building new knowledge, there are differences between them. The former focuses on the (socio-)cognitive conflict and the unfolding of natural dispositions, which result from interaction with the outside world, including the social world.^9,10^ The later, instead, focuses on the outside world itself, represented by the surrounding culture, paying special attention to competence differences with the subject.^7,11^

One particular area in which conflict brings about cognitive development is conceptual change.^12^ Conceptual change describes a diversity of changes, which occur in processes of learning and knowledge development.^13,14^ These include individual and social processes, and some involve radical conceptual transitions in which grounded conceptual models collapse and are replaced by new ones.^15,16^

From infancy, children have intuitive understandings of how the world works including, for example, elementary notions of physics, mathematics and social entities.^15,17^ These general intuitions, partly built on everyday experiences, often collide with scientific knowledge.^18–20^ The majority of researchers agree that they form coherent mental theories and models, which constrain interpretation of new data,^14,19,20^ in contrast to alternative knowledge-as-fragments views.^21^ In particular, some of these knowledge-as-theory approaches regard conceptual change as ranging from simple and easy enrichment of existing conceptual structures, to the more difficult radical revision of framework theories.^22^

Learning in science then may involve conceptual change. Therefore, these processes are difficult to attain,^23^ especially when they imply revision of framework theories,^22^ raising the need for specific teaching strategies.^20,24^ These gradual processes from intuitive understandings to more-advanced knowledge involve accommodating new information into existing conceptual structures, giving rise to alternative synthetic mental models constrained by children’s presuppositions.^25^ As a result, in educational settings, it is common to find children at different points along these developmental paths.^26^ This likely leads to situations of knowledge disparity, where different children hold substantially different theories about the same phenomenon.

When members of a group hold contrasting opinions about one matter, the arising socio-cognitive conflict presumably leads to processes of knowledge development and is of fundamental importance for conceptual change to occur.^27,28^ The problem of conceptual change has concerned not only cognitivism, but socioculturalism as well. Attempts have been made to reconcile the two^7^ and, although they differ on exactly how it is attained and have approached the topic at different times along their development history,^7^ they agree that social interaction is central in promoting these processes.

But simply putting two people together may not be sufficient, and the exact conditions that are effective remain to be identified.^29^ Debate and deliberation in small sets of adults result in a drastic increase in the quality of judgments made by groups,^5,30^ and this procedure seems to be appropriate for children as well, who are known to be able to both evaluate as well as to produce shareable arguments.^31^ During knowledge construction children can engage in collaborative argumentation, where individuals work together to construct and critique arguments, whereas remaining free to explore positions flexibly and to make concessions.^28,29^ This may promote conceptual and developmental changes in children,^10,28,32^ in part owing to the cognitive elaboration demands that argumentation requires.^28,33^ It is through this epistemic kind of conflict elaboration that positive cognitive outcomes would result, in contrast with other more relational kinds, which focus on individual confidence or assumed expertise.^6,11,34^ In fact, research seems to suggest that to induce conceptual change through peer collaboration, engagement in peer argumentation is required.^29^ Particularly, differences in scientific knowledge are especially elaborated through group decisions based on reasoning and argumentation, and plenty of evidence exists that arguing serves a function in learning elusive science concepts.^35^

However, there is also evidence indicating that social interactions may lead to irrational collective behaviors. They may cause informational cascades that lead to herding,^36,37^ or boost wide spreading of fake news^38^ or pseudo-scientific ideas,^39^ thus tempering their beneficial effects. Hence, whether interacting children with different views will generally progress (or not) towards a model based on reasoned and scientific evidence is an open question, which requires empirical examination. To explore this, we chose to study the domain of observational astronomy, cosmology, and world views (hereinafter, referred to simply as observational astronomy), a field where recent years have seen a rapid rise of pseudo-scientific thought, with international movements reinstalling ideas left behind centuries ago.^40,41^ Children hold a wide diversity of cosmologies and mental model representations of the earth, including its shape, where we live on it, and how it relates with the surrounding space.^42–44^ Many of these alternative models are inaccurate and arise along the different developmental paths through which children’s gradual conceptual change processes may occur.^22^ This lays a fertile field for socio-cognitive conflict studies in children, by leveraging knowledge differences naturally occurring within one same classroom. This domain of knowledge is also appealing to work with because of its rich network of concepts and conceptual relationships, and because the field itself has undergone radical restructuring in its historical development, which may lead to analogous changes in children as well,^20,26^ and makes it amenable for a quantitative analysis of conceptual development.

Although evidence for collaboration as a tool for intellectual gain does exist, there are few rigorous experimental studies that compare groups against individuals engaging in a comparable task, and which demonstrate greater gains for the group condition. This is even more so for ill-structured tasks, which lack a single correct answer.^32^ The main goal of the present work is to understand collective construction of knowledge, and the corresponding developmental processes within each individual, when children hold and share arguments in favor of different conceptual models. To this aim, we measured and quantified the mental models of the earth of a group of children before and after peer interaction, and against those of a control group, in which children reviewed the material alone, without interaction. Peer interaction consisted in a collaborative work in which children were asked to express a consensus—through a drawing—of several geographic and astronomic features, which probed their understanding of our planet. Before this interaction, in a first interview, we assessed the degree of knowledge of each child through both factual and generative questions, and then grouped them in dyads of one Less knowledgeable (L) and one More knowledgeable (M) child. Given that both children in each dyad had given different responses in Interview 1, our experimental design allowed us to quantify: (a) whether children change their conceptual understanding of the earth after a collaborative work, (b) if the mental representations of the two children in each dyad become more similar after interaction, and (c) whether this change reflects a progression towards a more advanced representation of knowledge, in accordance with our current physical and cosmological understanding (e.g., that the earth is spherical and not flat).

## Results

### Changes in children’s knowledge level after interaction

In this work, children’s knowledge was assessed before peer interaction to evaluate their initial mental models. This information was then used to pair children with contrasting views in dyads, so that one member had a better understanding (*Child M*: More knowledgeable) than the other (*Child L:* Less knowledgeable). Next, both members of each dyad worked together in a collaborative argumentation task. Finally, all children’s mental models were assessed again after interaction.

**Fig. 1:**
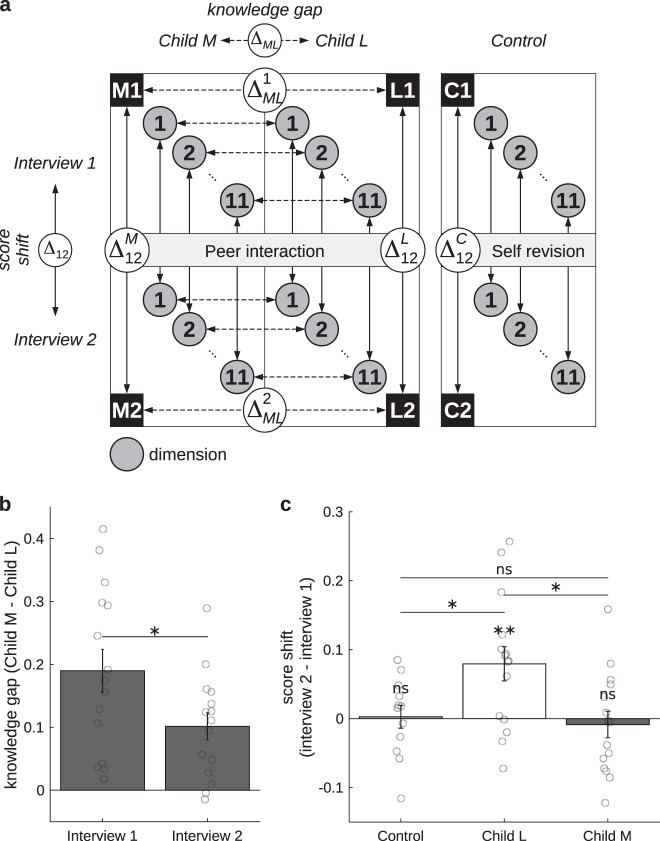
Knowledge level changes after interaction. **a** Knowledge level was scored in 11 dimensions per session. Differences between interviews of one child (shift, *Δ*_*12*_) and between members of a dyad (gap, *Δ*_*ML*_) were calculated on a per dimension basis and then averaged. ${\Delta _{{\mathrm{12}}}}$: knowledge level shift between Interviews 1 and 2, for Children M (${\Delta _{{\mathrm{12}}}^M}$), L (${\Delta _{{\mathrm{12}}}^L}$) and control (${\Delta _{{\mathrm{12}}}^C}$); ${\Delta _{ML}}$: knowledge level gap between Children M and L, in Interviews 1 (${\Delta _{ML}^{\mathrm{1}}}$) and 2 (${\Delta _{ML}^{\mathrm{2}}}$). M1/M2, L1/L2, C1/C2: more knowledgeable (M), less knowledgeable (L), and control (C) children in Interview 1 or 2. **b** Average knowledge level gap between children in each dyad was significantly smaller after interaction than before. **c** Shift in knowledge levels. Children L significantly increased their knowledge levels. In contrast, no significant gains or losses were found for either controls or Children M, between whom no significant differences were found either. (*p < 0.05, **p < 0.1, ***p < 0.001, ns: non-significant. Error bars represent SEM, and circles are individual data points)

Children’s conceptual knowledge about the earth was quantified by mapping their verbal, drawn and gestural responses into 44 traits that encode different elements of knowledge (shape of the earth, how it is positioned relative to other astronomical objects, location of the sea, the clouds and people, etc). These 44 traits were collapsed into 11 dimensions, which provide a summary of children’s understanding of the representation of the earth (see Methods and Supplementary Tables 2 and 3 for full details on the coding scheme and how it is reduced to relevant dimensions). The difference in knowledge level, either between two members of a dyad (hereafter knowledge level gap: Δ_ML_) or between two stages (before and after peer interaction/self revision) for a given child (hereafter, knowledge level shift: Δ_12_), was calculated for each dimension, and then averaged (see Fig. 1a).

On average, the knowledge level gap between both members of a dyad (Δ_ML_) was narrowed after peer interaction, as found with a paired-samples *t* test, *t(14)* = 2.75, *p* = 0.016, *d* = −0.61, 95% CI (−1.35, −0.12) (Fig. 1b). This could have been caused by (a) an increase in knowledge level of Children L, which would suggest that a collaborative work is beneficial for conceptual change to occur, (b) a decrease in knowledge level of Children M, which would imply that more advanced mental models are called into question owing to interaction with a less-knowledgeable interlocutor, or (c) a combination of both. To discriminate between these possibilities, we analyzed the knowledge level of each child before and after peer interaction. These analyses showed a significant positive knowledge level shift for the less-knowledgeable dyad members (Δ^L^_12_), *t*(14) = 3.21, *p* *=* 0.006, *d* = 0.83, 95% CI (0.23, 1.41). By contrast, we did not observe a significant knowledge level shift when differences were measured for the more knowledgeable dyad members (Δ^M^_12_), *t*(14) = −0.45, *p* = 0.662, *d* = −0.12, 95% CI (−0.62, 0.39). These results indicate that the reduction in the knowledge level gap was in fact owing to an increase in knowledge level of Children L, who before the interaction held a less advanced mental representation of the earth.

To evaluate whether this knowledge level shift found for Children L could simply be explained by the fact that they had an opportunity to review the topic and/or that they were interviewed twice, we performed a control experiment. We measured knowledge level shifts of children who also reviewed the topic, but who did not participate in collaborative work. Results showed no increase in knowledge level in the control group (Δ^C^_12_), *t*(11) = 0.16, *p* = 0.875, *d* = 0.04, 95% CI (−0.53, 0.60) (Fig. 1c). This strongly suggests that the results found in our main groups cannot be accounted for by a mere repetition of the questionnaire or by the effect of reviewing the topic with a drawing exercise.

In addition, a one-way analysis of variance (ANOVA) showed a significant group effect on knowledge level shift Δ_12_ between interviews, *F*(2, 39) = 5.42, *p* = 0.008, *η*² = 0.211, with post hoc Tukey’s HSD test showing significant differences between Children L (Δ^L^_12_: *M* = 0.08, SEM = 0.02) and both controls (Δ^C^_12_: *M* = 0.00, SEM = 0.02, *p* = 0.042) and Children M (Δ^M^_12_: *M* = −0.01, SEM = 0.02, *p* = 0.011). Controls and Children M did not significantly differ from each other (*p* = 0.928).

Because dyads had to be formed with one child with a lower score than the other to evaluate directionality of knowledge changes, scores were not equally distributed between groups in Interview 1. Particularly, mean score for Children L was the lowest. We wondered then if unequal distribution between control, Child L and Child M groups could have been the reason behind Children L’s knowledge level shifts. Indeed, a one-way ANOVA found significant differences in mean Interview 1 (initial) scores between the three groups, *F*(2, 39) = 10.23, *p* = 2.69E−04, *η*² = 0.327. Hence, it is possible that the effect observed in Children L and not in the control group would be solely a consequence of their initial scores. We discard this possible confound in two different ways: first, while post hoc comparisons using Tukey’s HSD test found, as expected, significant differences between Children L (*M* *=* 0.53, SEM *=* 0.04) and M (*M* = 0.72, SEM = 0.02, *p* = 1.68E−04), no significant differences were found between controls (*M* = 0.61, SEM = 0.03) and either Children L (*p* = 0.169) or M (*p* = 0.053).

To further verify that Children L’s lower scores in Interview 1 were not the cause of their larger shifts, a restricted analysis was conducted, which considered only participants with Interview 1 scores between 0.45 and 0.75, a score interval within which both controls and Children L were similarly distributed. As observed before, employing the entire data set, Children L (*n* = 12) showed significant knowledge gains, *t*(11) = 2.71, *p* = 0.020, *d* = 0.88, 95% CI (0.20, 1.54), whereas controls (*n* = 11) did not, *t*(10) = 0.08, *p* = 0.935, *d* = 0.02, 95% CI (−0.57, 0.61). This shows that the significant increase in Children L’s scores was not owing to lower Interview 1 scores, but rather owing to engagement in a collaborative task with a more knowledgeable peer.

Altogether these results show that the collective conceptual representation of the earth held by children in a dyad progressed. This was the result of children with a less-advanced mental representation approaching a more sophisticated mental model, whereas we found no evidence to support that knowledge level changed for the more knowledgeable children.

### Changes in children’s knowledge similarity after interaction

The results described above showed that knowledge level improves in children after interaction with a more knowledgeable peer. This has two possible explanations. A purely constructivist possibility is that the process of reflection with a more knowledgeable peer made children question—in a broad and general manner—aspects of their own conceptual representations. Another possibility is that the less-knowledgeable children adopted specific facts and elements transmitted from the mental representations of their peers.^45^ If the latter was true, Children L would not only be expected to have increased their knowledge levels, but also to express representations closer to their more knowledgeable peers. To examine this, we quantified and measured the similarity in a multi-dimensional knowledge space between both children in each dyad, before, and after peer interaction.

**Fig. 2:**
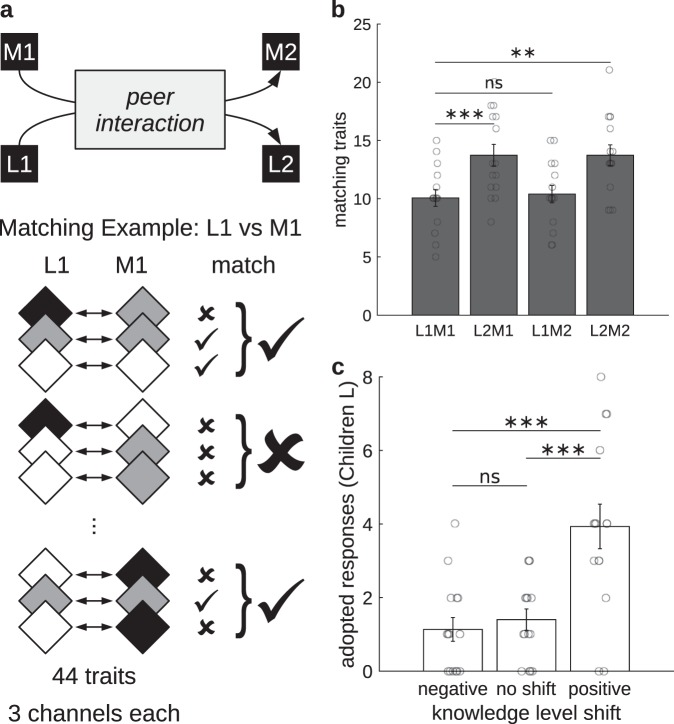
Knowledge similarity. **a** Responses of Children L and M are coded in Interviews 1 (L1 and M1) and 2 (L2 and M2). To assess knowledge similarity, coding of each trait in each channel is compared. In the example, Child L’s and Child M’s Interviews 1 (L1M1) are compared. A trait is said to match if it was given the exact same code in both cases (both children or both interviews) in at least one channel. **b** Matching of traits within dyads. Children L and their peers matched in significantly more traits after (L2M2) than before (L1M1) interaction. Particularly, Response Adoption (i.e., modification of children’s responses to resemble those of their partners) was significant for Children L (L2M1–L1M1), whereas not for Children M (L1M2–L1M1). LxMy: number of traits matching between Child L in Interview x and Child M in Interview y, with x/y: 1 or 2. **c** Knowledge shifts led by Response Adoption. Adopted responses are defined as traits matching between a child’s Interview 2 and her partner’s Interview 1, whereas not between them before interaction in Interview 1. Here, adopted responses were divided into groups of positive, negative, or no knowledge level shift. Responses adopted from their partners led to significantly more knowledge gains than either losses or no changes in Children L. (*p < 0.05, **p < 0.01, ***p < 0.001, ns: non-significant. Error bars represent SEM, and circles are individual data points)

Knowledge similarity can be measured as the inverse of a Hamming distance between two vectors of conceptual representation, measuring in how many of the 44 traits the two vector representations are equal. We define two traits to match in any vector comparison (either between two children, or between two interviews of the same child) if they were assigned exactly the same predefined code in at least one of the three possible channels: verbal, drawn or gestural (Fig. 2a). For example, if trait “Where to look to see the Earth” was coded “Upwards or at the sky” in the gestural channel for both children in a dyad (or for both interviews of a child), that trait would be considered to match between children (or between interviews). But not so if it was coded “Upwards or at the sky” for one child (or interview), and “Towards or in space” for the other.

We first compared traits matching between children in a dyad before (L1M1) and after (L2M2) peer interaction (see Fig. 2a). A paired-samples *t* test showed that the mean number of traits matching between peers significantly increased after the collaborative task, *t*(14) = 3.41, *p* = 0.004, *d* = 0.99, 95% CI (0.23, 1.75) (Fig. 2b).

Given that not all traits can be scored to quantify knowledge level (see Supplementary Table 3), this greater similarity could be accounted for either by (i) the less knowledgeable (L) children approaching the mental representations of their more knowledgeable (M) peers; (ii) the other way around, with the more knowledgeable approaching the less knowledgeable; or (iii) both ways, meeting at some middle ground. To discriminate between these alternatives, we measured the number of responses that children adopted from their peers. We refer to this measure as response adoption: *RA*^*L*^ *=* *L2M1–L1M1*, for Children L, and *RA*^*M*^ *=* *L1M2–L1M1*, for Children M; where L2M1 and L1M2 are the mean number of traits matching between children (L or M, respectively) in Interview 2 and their partners in Interview 1, and L1M1 is the mean number of traits matching between children and their partners in Interview 1 (see Fig. 2a). Paired-samples *t* tests showed that response adoption was found significant only for Children L, whereas not for Children M, *t*(14) = 4.91, *p* = 2.32E−04, *d* = 1.51, 95% CI (0.70, 2.32), and *t*(14) = 0.42, *p* = 0.680, *d* = .11, 95% CI (−0.61, 0.83), respectively (Fig. 2b). Subsequent analysis showed that Children L’s response adoption from their M peers did not rely (or did not only rely) on traits or trait channels not used before interaction, but rather on response modifications following the collaboration task (see Supplementary Table 4).

The results of the first section showed that the less-knowledgeable children broadly increased their knowledge levels, as quantified by a scalar which measures whether knowledge approaches scientific explanations of the earth. In this second section, we zoomed in on this result by measuring knowledge similarity in a high-dimensional space, and showed that Children L and M resemble each other more after peer interaction, due to Children L adopting their peers’ responses, and not the other way around.

### Response adoption and knowledge level shifts

Results so far show that, after collaborative peer interaction, (i) less-knowledgeable children increased their knowledge levels (Fig. 1c), and that (ii) they adopted responses from their more-knowledgeable peers, approaching their mental representations. Given that during interaction children could exchange information freely, response adoption could either have led to incorporation of correct, incorrect, or equivalent information; i.e., positive, negative, or no knowledge level shifts, respectively. To verify that their knowledge levels actually increased because of correct response adoption, the nature of these response changes was analyzed.

A response adopted from a peer is defined as a trait which matched between a child’s Interview 2 and her partner’s Interview 1 (i.e., between L2 and M1, or between M2 and L1), whereas not matching between them before interaction in Interview 1 (i.e., between L1 and M1; see Fig. 2a). Only for Children L, responses adopted from their peers significantly led to positive knowledge level shifts. A one-way ANOVA found significant differences between the average number of adopted responses leading to either positive, negative or no knowledge level shifts, *F*(2, 42) = 12.94, *p* = 4.18E−05, *η*² = 0.37 (Fig. 2c). Post hoc comparisons using Tukey’s HSD test showed that the number of adopted responses leading to positive shifts (*M* = 3.93, SEM = 0.61) was significantly larger than those leading to either negative (*M* = 1.13, SEM = 0.32, *p* = 1.09E−04) or no shifts (*M* = 1.40, SEM = 0.29*, p* = 4.31E−04). Conversely, no significant differences in the number of adopted responses leading to positive, negative or no shifts were found for Children M, *F*(2, 42) = 0.67, *p* = 0.518, *η*² = 0.03. Similar results were obtained after dividing the counts of adopted responses by the total number of traits coded, to prevent participants with more coded traits from biasing the results (see Supplementary Table 4).

These results suggest that Children L adopted significantly more correct responses from Children M, leading to positive knowledge level shifts or gains. On the other hand, Children L’s responses adopted by Children M led to no significant knowledge level changes overall.

### Summary

In short, our results show that, when presented with a collaborative task, children with less-advanced views of the earth significantly increased their knowledge levels after a brief but significant interaction with a peer who presented a more-sophisticated mental model. These gains can be explained by the less-knowledgeable children adopting their peers’ responses. Overall, these results suggest that peer tutoring, as a special case of collaborative argumentation, helps solve knowledge differences between children and contributes to collective construction of knowledge, by pushing the less-knowledgeable forward through conceptual change, instead of by simply bringing them to some middle ground.

## Discussion

The main goal of the present work was to understand collective construction of knowledge, and the corresponding developmental processes within each individual, particularly when children hold and share arguments in favor of different conceptual models. We hypothesized that, socio-cognitive conflict between children with contrasting views of the earth, elaborated through collaborative argumentation, would help promote collective construction of knowledge, boosting the otherwise slow occurrence of conceptual change. To explore this possibility, we presented second graders with a collaborative peer interaction task. In line with previous proposals to reconcile cognitivist and socioculturalist views on conceptual change,^7^ this task was based on the neopiagetian socio-cognitive conflict paradigm,^10,12,34^ whereas at the same time it included neovygotskian knowledge level and role asymmetries. These asymmetries, although implicit, link our task with peer tutoring approaches, where acquisition of knowledge arises through active helping and supporting among peers with similar but different knowledge levels, from similar social groupings (close to each other in age, ability, knowledge level, and other characteristics), and who are not professional teachers.^23,46^ We interviewed 7-year-old children and then paired them in dyads where one of the members had a more-advanced knowledge (Child M) than the other (Child L) in the field of observational astronomy. This knowledge asymmetry was undisclosed to the participants. During peer interaction they had to complete a drawing task, for which they had to agree on how to draw a series of concepts related to different aspects of our planet. Afterwards, a second interview was conducted to evaluate possible changes in children’s mental models. Our approach focused on verbal, drawn, and gestural responses to better probe children’s knowledge,^47–50^ and was laboratory-based, with sessions taking place outside the classrooms, in a controlled room provided by the school.

### Collaborative peer interaction increases knowledge levels

Children’s knowledge assessment showed the wide diversity of mental representations expected from previous works,^19,26,42–44,51^ This provided the appropriate framework to conduct our research, as it enabled us to induce socio-cognitive conflict by leveraging knowledge differences naturally occurring in the classroom, rather than by introducing them artificially.

First, when children engaged in collaborative interactions with a more knowledgeable peer, they significantly increased their knowledge levels by changing their responses to resemble those given by their partners. In contrast, these knowledge level increases were absent when participants reviewed the material alone without interacting with a peer. These findings strongly suggest that knowledge construction was promoted by peer interaction, and not by simple self revision of the concepts or interview repetition.

These results are consistent with true conceptual understanding, as opposed to simple peer influence and repetition. On the one hand, the post-test was analogous, but not identical, to the peer interaction or self revision stage. In fact, not only were they framed differently (a questionnaire vs a drawing task), but moreover, the social context was different as well (adult-guided vs collaborative or solitary). This isomorphism, from peer interaction or self revision stage to post-test, would entail application of the same principles or concepts for the solution of different tasks^45^; this in turn would imply certain transference, making it less likely that children were simply imitating their peers without true understanding.^45^ On the other hand, knowledge changes were not evaluated immediately after peer interaction or self revision stage, but rather a delay of 1 week on average was left; the fact that knowledge changes were preserved after this incubation period is compatible with true understanding as opposed to simple repetition.^52^ Nevertheless, greater differences and longer delays between peer interaction and post-test could help further disentangle these possibilities.

In accordance with previous results in the field of peer tutoring, we expected that the more knowledgeable children would also benefit from collaborative interaction,^46,53–56^ especially considering that the small knowledge level gap with their less knowledgeable peers would provide them with cognitive challenges as well. However, we found no evidence to support this. One possible explanation could be that, whereas the initial score for the less-knowledgeable children in Interview 1 was variable, from low to medium, the more knowledgeable children had higher initial scores. This could have created a ceiling effect, limiting the observation and quantification of knowledge gains. In addition, whereas average gains were null for Children M, there was a marginal trend toward knowledge gains in those who interacted with the most knowledgeable of Children L (Supplementary Figure). This trend may suggest that Children M could benefit from collaborative argumentations if greater cognitive challenges were provided through interactions involving smaller knowledge differences within the dyad.^46^

Although this result is marginal and, hence, we cannot conclude that Children M may benefit from peer interaction, we did not observe knowledge degradation owing to interaction with a less knowledgeable peer. This has important practical implications, since in the educational community a common apprehensiveness is what would happen in natural peer interactions when knowledge may be labile for both. In our study, social disagreement and argumentation in children led toward construction of more-advanced knowledge: the more knowledgeable children helped promote conceptual change in the less knowledgeable, without detrimental effects on their own understanding. From a knowledge-as-theory perspective, this finding is compatible with the Piagetian idea of concepts becoming necessary truths once fully understood,^9^ and helps dispel the notion that peer interaction may be counterproductive for children collaborating with less knowledgeable peers.^57^

### Collaborative peer interaction and agreement

When people in a group hold different opinions and try to reach an agreement to find the “right” one, different strategies may be involved. One possibility is to select an average combination of all choices. It is well established that, depending on the nature of the situation, choosing the average response from a large group of independent people is better than choosing any of the individual responses, a phenomenon popularly known as wisdom-of-crowds.^1^ However, losing independence among the individual responses is known to break the wisdom-of-crowds effect.^2^ In fact, situations in which mass behavior is detrimental, such as in herding,^36,37^ are widely common.

Conversely, interaction among members of a group, instead of simply witnessing each other’s responses, may boost group over individual performance. For example, dyads perform better than individuals when they are allowed to reach consensus through free interaction in low-level perceptual decision-making tasks, presumably by sharing their confidences about their responses.^3^ Accordingly, Koriat has even suggested that the interaction can be entirely circumvented by simply choosing the most confident response.^4^ A similar positive deliberation effect, presumably mediated by confidence and expertise-assumption heuristics, was found in the estimation of general knowledge quantities, both in the group response, as well as in individual responses given after interaction.^5^ Nevertheless, when group decisions are based on reasoning, it has been suggested that communication is not focused on individual confidence or assumed expertise, but rather on shareable arguments.^6^

Our results showed that the knowledge gap narrowed because the less knowledgeable children increased their knowledge level, and not because the more knowledgeable decreased theirs. This suggests that children did not reach an average agreement between their original points of view. Instead, these results strongly suggest that agreement was reached at some point skewed toward the more knowledgeable member of the dyad. We pose that this bias could have been driven by different heuristics, such as (a) perceived confidence of the partner on his/her point of view, (b) authority cues possibly provided by the asymmetrical role assignment,^58,59^ or even by (c) arguments provided by either side. The fact that knowledge level increases were preserved after a delay of one week on average, is compatible with socio-cognitive conflict being elaborated through collaborative argumentation.^52^ Children’s engagement in the peer interaction task, the fact that they had to give only one joint answer,^28^ together with knowledge level asymmetry, may be some of the reasons why free collaboration could have spontaneously derived into collaborative argumentation, without explicit instruction. Detailed analysis of the peer interactions themselves^7,60^ may further confirm that socio-cognitive conflict was in fact elaborated through collaborative argumentation, and help better describe the conditions under which free collaboration promotes conceptual change.

In this work, we also introduced a slight role asymmetry by systematically giving only to the more knowledgeable member of the dyad the instructions of what they were supposed to do together. Although this role asymmetry was small and it did not explicitly imply that one participant knew more than the other, or that one should teach the other, we cannot disregard this point and future research should address it.

### From Aristotelian to Copernican thought

It is widely agreed that children build relatively coherent theories of how the world works, which they enrich and revise in face of the interaction with their surrounding and often conflicting culture.^14,17,19,20,61^ In this study, we evaluated children’s knowledge using this knowledge-as-theory perspective. In particular, we assumed the broad Framework Theory approach to conceptual change, which defines it as any knowledge change that occurs during processes of learning and development, ranging from the simple enrichment of existing conceptual structures, to the more difficult radical revision of framework theories.^14,22^ Some authors, on the other hand, argue for a knowledge-as-fragments view instead.^19^ According to this view, children have neither prior conceptions nor misconceptions, but rather are theory free, their knowledge being fragmented rather than coherent,^21,62^ and with conceptual change occurring through gradual enrichment by accumulation of fragments from the culture.^63^ Our results may have a say in this framework as well, given that the knowledge level shifts we found are independent of whether children have coherent or fragmented prior knowledge. However, the vast majority of researchers in the field of observational astronomy endorse the knowledge-as-theory approach.^51^

**Fig. 3:**
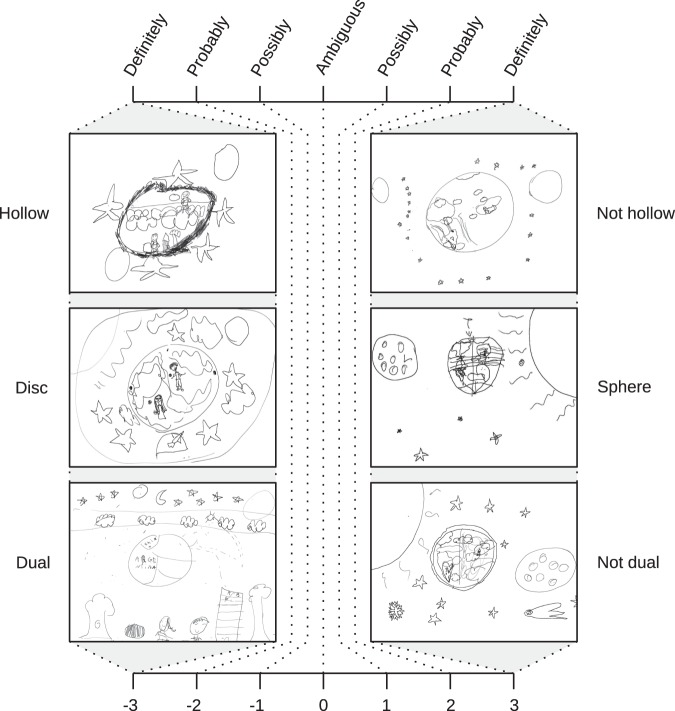
Coding scheme 1: coarse-grained scheme. Participants were first scored with Coding scheme 1 to pair them in dyads for peer interaction. This scheme consisted of three axes, each one scored in a 7-point Likert-type scale: hollow/not-hollow, disc/sphere and dual/not-dual. Representative drawings of extremes responses for each axis are shown. This scheme was based on previously described children’s mental models: (1) the rectangular (flat) earth: with people living on flat ground which extends all the way down below the earth; (2) the disc earth: where flat ground is shaped as a disc; (3) the dual earth: with a flat earth on which people live and another earth suspended in space; (4) the hollow sphere earth: where the earth is suspended in space and people live on flat ground deep inside it; and (5) the flattened sphere earth: with gravity holding people on flattened areas at the earth’s top and bottom^26,42–44^

From this perspective, the process of conceptual change from an initial mental model of a flat planet to that of a mature understanding of a spherical earth occurs through the gradual revision of framework theories’ presuppositions. This is a slow and gradual process, during which children try to accommodate the information they receive—from everyday experience—into their existing conceptual structure, giving rise to alternative mental models constrained by children’s presuppositions (Fig. 3). The simplest models occur at younger ages, whereas the more sophisticated and the scientific ones are found in older children.^14^ The process of conceptual change involves a developmental shift in categorization of the earth to reconceptualizing it as an astronomical object.^14,64^ This process is analogous to how scientific theories change throughout time, as is particularly the case with astronomy.^65^

Although children’s learning pathways are complex and the development of their conceptual understandings is not linear or hierarchical,^19^ a general path can be traced. At the beginning of the conceptual change path, children’s concept of the earth is constrained by the presuppositions of their naive framework theory of physics. For instance, they assume that people live on flat ground, and around the age of four months they grasp the notion that unsupported objects fall in a downward direction.^66^ These presuppositions limit their interpretation of observations and cultural information,^20^ and give rise to a set of beliefs or specific theory about the earth.^14,22^ Usually next, as children continue to accommodate the information they receive from the environment, dual-earth synthetic models emerge, given that they do not require revision of presuppositions, but rather simple addition of novel information: by accretion of new scientific information to their existing conceptual structures, they assume that adults refer to a different object when talking of a round earth in space. Then, later along the developmental path, children revise their presupposition that unsupported things fall, allowing them to conceptualize the earth as an object suspended in space. However, the fact that this presupposition still holds for objects (and people) on the earth’s surface, constrains the types of suspended-earth models possible, giving rise to models of flat disc, or hollow sphere with people living on flat ground inside the sphere. Finally, after reinterpretation of this and flat-ground presuppositions, children are able to conceptualize the earth within the explanatory framework of solar/astronomical objects as a sphere with people living outside and all around held by gravity.^26,51^

A complementary analysis (see Supplementary Discussion), in which related knowledge traits were grouped into three themes, revealed that the traits behind the knowledge level gains reported were mainly those referring to aspects related to the placement of objects on the surface of the planet. These are, in turn, associated with the dual/not-dual axis of the coarse-grained Coding scheme 1 (see Methods section). This result suggests that peer interaction may have favored revision of dual-earth models by the less knowledgeable, which is the simplest and first alternative synthetic model along children’s conceptual change path. Therefore, the knowledge changes we observed not only indicate that the less-knowledgeable children benefited from peer interaction by increasing their knowledge level instead of averaging it with their partners’, but also that interaction may have promoted this by helping children reinterpret their presupposition that the earth needs to be supported, switching from a dual to a non-dual mental model. This is compatible with deep conceptual development resulting from interaction, rather than simpler learning of specific facts, and would be the first step toward reconceptualizing the earth as an astronomical object.^22^ This ontological recategorization, which usually occurs between around 3rd and 5th grade,^25^ would precede children’s full understanding of the earth as a spherical planet.^14^ In other words, the more-knowledgeable peer would lead her partner to understand what she is closest ready to understand: that the earth we live on is suspended in space. This suggests an inviting parallelism with Socrates’ statement during his dialog with Meno, in which he holds that he is not teaching when asking his young pupil about doubling the area of a square, but rather that the pupil knew the answer, but he did not know that he did.^67^ In a way, peer interaction may catalyze conceptual change, which would occur anyway, although more slowly. Focusing on a narrower set of concepts and conceptual relations could help better identify the exact conceptual change processes promoted by these interactions.

### Final remarks

Our results show that engagement in a simple collaborative argumentation task may catalyze the laborious revision of early entrenched presuppositions. Particularly, peer interaction, between children with different mental models in the field of observational astronomy, helped promote processes of conceptual change towards the most advanced models, fostering collective construction of knowledge. Here, we provided evidence that interaction with a more knowledgeable same-age peer helped children modify their notion of the earth and blend two extreme visions of the planet: that of an object suspended in space, and that of a seemingly flat surface on which they live. Moreover, this occurred without negatively impacting the more knowledgeable children.

In line with previous proposals to reconcile cognitivist and socioculturalist approaches to conceptual change, our approach would fulfill three requirements proposed for conceptual leap to occur.^7^ Knowledge-level asymmetries would have provided alternative discourses to the less knowledgeable children. Moreover, the fact that these asymmetries were implicit may have translated into higher levels of equality,^23^ which in turn may have helped make the conflict overt, rather than having the more knowledgeable children impose their knowledge on the less knowledgeable. Finally, implicitness of knowledge level and role asymmetries could also have brought peers to a closer position and kept levels of mutuality high, typical of peer tutoring interactions,^23^ which probably helped the less-knowledgeable to position as capable of taking the necessary conceptual leap.

Altogether, the present work contributes to identifying individual and collective aspects of social interactions, which favor knowledge transfer, and to understanding the basic mechanisms through which these interactions mediate agreements and, ultimately, social construction of knowledge. Future research including younger and older children, from a wider variety of socioeconomic and cultural backgrounds, would help generalize our results to a wider population.

Standing on the shoulders of previous identification of children’s concepts about the earth, the present work focuses, in turn, on the learning processes in this domain, and on how to support them.^68^ This would eventually allow a more-immediate interpretation by and transfer to education, pedagogy and public policy design. Argumentation is key to learning science. However, science education is notable for the absence of arguments,^35^ and specific teaching practices are needed to help promote the corresponding conceptual change processes involved,^20^ which instructor explanations often fail to address.^24^ Our study provides laboratory-based empirical evidence for alternative, more-efficient ways to foster these processes, by leveraging knowledge differences naturally found in the classroom to elicit socio-cognitive conflict presumably elaborated through collaborative argumentation. It therefore provides empirical evidence to inform classroom-based research on instructional strategies in the cognitive conflict paradigm of conceptual change.^12^

Finally, new research may address how our results could translate into strategies to deal with the growing spread of pseudo-scientific ideas about the shape of the earth.^40,41,69–71^ Seriously acknowledging misconceptions and knowledge differences, and promotion of scientific knowledge by leveraging peer elaboration of socio-cognitive conflict through collaborative argumentation, might help pave the way away from today’s science denial crisis.

## Methods

### Participants

A total of 46 second graders (23 female) from a private medium- and high-SES bilingual school participated in the study. Data from four children (one female) had to be discarded due to technical problems. Ages of the 42 remaining children ranged from 7 years to 8 years and 3 months, with a mean age of 7 years and 6 months. Sessions took place outside of the classroom in a quiet room provided by the school for the purpose of this study.

All children’s parents or legal guardians gave signed voluntary consent previously authorized by an Ethical Committee - Comité de Ética de la Dirección de Investigación del Centro de Educación Médica e Investigación Clínica “Norberto Quirno” (CEMIC), Unidad Asociada del CONICET, Protocol N° 683.

### Software and equipment

All sessions were registered using two Logitech HD Pro C920 cameras with integrated microphones. Drawings were made on a Wacom screen using pressure and tilt sensitive pens. Drawing canvas was provided by GIMP software. Video, audio and screen were simultaneously recorded using VideoLAN’s VLC software on a computer running Linux Mint 17.

### Experimental design

Children were interviewed twice to assess their mental models of the earth before and after interaction in dyads. A first coarse-grained coding scheme was used to rapidly evaluate children after Interview 1, and to form dyads of children with different knowledge levels. A second fine-grained coding scheme was used later to assess knowledge changes between interviews.

Thus, each participant engaged in three separate sessions: (a) Interview 1 (pre-test), (b) peer interaction/self revision stage, and (c) Interview 2 (post-test):

(a) Interview 1: All participants were interviewed individually by a research assistant following a tightly scripted questionnaire. The questionnaire was designed based on the work of Vosniadou and Brewer.^26^ It consisted of 50 questions aimed to reveal the conceptual mental models of the earth held by the participants (the complete questionnaire is available as Supplementary Table 1). Questions explored topics such as the shape and placement of astronomical entities (planet Earth, moon, stars, sun), sky items (sky, clouds) and earth objects (people, houses, trees, countries, sea), as well as the exploration of the planet’s surface (straight-line walking and edges). As children often change their responses in conditions of repeated questioning,^72,73^ the precise strategy for follow-up and additional questions was predefined to avoid interviewer bias. Participants were familiarized with the drawing screen before Interview 1, and they were free to draw, speak, and gesticulate to answer all questions.

Coding scheme 1 (coarse-grained scheme): To make sure that the mental models revealed in Interview 1 changed as little as possible before peer interaction/self revision, time between these two stages had to be short. To assure that we could assess children’s knowledge and form the dyads in only 2 days, we used a coarse-grained coding scheme, referred to as Coding scheme 1, that allowed us to rapidly evaluate children. The responses of each child were coded in three axes: hollow/not-hollow, disc/sphere and dual/not-dual, based on mental models previously described by Vosniadou and Brewer.^26^ We considered verbal, drawn and gestural responses, and each of the three axis was scored in a 7-point Likert-type scale ranging from −3 to + 3. These scores were assigned by the first author, who watched each interview’s video and gathered relevant evidence to decide sign (+/−) and value (1–3) of each axis independently. Negative scores indicated that the children’s responses revealed hollow, disc, or dual models of the earth, respectively. Conversely, positive scores were used for participants giving responses compatible with not-hollow, sphere or not-dual models. A zero value in a given axis indicated that responses revealed ambiguous descriptions, and values closer to + 3 or −3 reflected that the children’s responses clearly corresponded to one of the two opposing extremes of the axis. For example, answering “upwards or through a telescope” to the question Q05 “Where do we have to look at to see planet Earth?” was taken as evidence in favor of a dual model and hence shifted the score in the dual/not-dual axis towards −3. Instead, answering “downwards” and/or pointing to the ground shifted that score towards + 3. Representatives of axis extremes are shown in Fig. 3. Scores in each axis were then linearly normalized to a 0–1 range.

It is important to note that this coarse-grained coding scheme was only used to pair children in dyads. We later confirmed that it is proper approximation for a second fine-grained coding scheme assigned by a blind research assistant and later used for analysis (see Coding scheme 2 below).

Using the coarse-grained coding scheme, participants were grouped into same-gender dyads, so that in each dyad one member had a better understanding (Child M, More knowledgeable) than the other (Child L: Less knowledgeable), meaning that she had a higher score for at least one of the coding scheme’s axes, and equal or higher for the other two. Fifteen of these dyads were randomly chosen to be part of the peer interaction group. These were revised and approved for interaction by the class teachers, based on their everyday experience at school. The remaining dyads were dissolved, and its 12 members assigned to the self revision control group instead. This procedure was used to assure that the members of the peer interaction and self revision groups were randomly selected.

(b) Peer interaction/self revision stage: Both children from each dyad were asked to work together. Child M was brought first to the room, was given a booklet and was told that she had to explain to her partner that they would have to work together and make one drawing depicting all terms written in the booklet. Then, Child L came into the room and, after reminding Child M to explain the task to her partner, the assistant left the room. 45 s later she came back, handed the drawing pen to Child M and left again until the children reported they had finished.

The booklet had the following 14 terms, one per page: “planet Earth”, “moon”, “stars”, “sun”, “sky”, “clouds”, Child M’s name, Child L’s name, “house”, “tree”, “Argentina”, “China”, “person who lives in China”, “sea”. The research assistant emphasized to Child M that it was important to reach an agreement before drawing each element of the booklet. All these terms had been discussed during Interview 1.

The 12 children in the control group had the same task of making a drawing that included all the terms in the booklet, but they performed this second session alone. In these cases, Child M’s and Child L’s name in the booklet were substituted for control child’s name and “a friend”, respectively. On average, 10 days passed between Interview 1 and peer interaction/self revision stage (min = 3d, max = 17d).

(c) Interview 2: All children then went to a third session in which they were asked the same questions as in Interview 1, following exactly the same procedure. This interview was conducted by a different interviewer. On average, 7 days passed between peer interaction/self revision stage and Interview 2 (min = 3d, max = 11d), and 17 days between Interview 1 and Interview 2 (min = 8d, max = 24d).

**Fig. 4:**
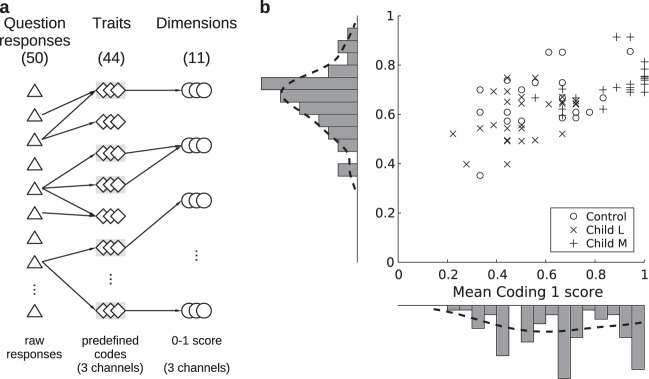
Coding scheme 2: fine-grained scheme. **a** Coding scheme. Responses to questionnaire questions are used to code 44 knowledge traits in three channels each. Some of these traits (boxed in gray), with predefined codes at two or more levels of knowledge, are scored from 0 to 1. Finally, to prevent some aspects of knowledge from being over- or underrepresented later in score differences, scored traits belonging to similar topics are combined into 11 dimensions and their scores averaged. **b** Comparison with Coding scheme 1. We compared Coding scheme 1 used to form dyads and Coding scheme 2 used for data analysis. Each point represents one participant’s interview, with its Coding 2 or mean dimension score and its Coding 1 or mean axis score. A strong correlation between scores from both coding schemes was found. Histograms next to each axis represent score frequencies, with kernel density smoothing function fit in dashed lines

Coding scheme 2 (fine-grained scheme): One of the main aims of this work was to investigate how conceptual representations of the earth changed after peer interaction, i.e., between the first and the second interviews. However, statistical comparisons with Coding scheme 1 were not possible because children’s responses saturated the scale in this coarse-grained scheme (i.e., many children had maximal values) and their distribution was not normal (see Fig. 4b).

To overcome this issue, we re-coded the responses to the questionnaire using a more refined, higher resolution, and objective scale. We identified 44 different topics, henceforth referred to as traits, which could be evaluated in a quantitative manner from children’ responses. These traits referred to different forms of knowledge about the earth, from geographical to astronomical, such as where to look at to see the earth, what the location of China is relative to Argentina (which were chosen because they are close to antipodes), the position of the moon relative to the earth and to the sun, or the location of the clouds relative to the sky. A series of codes were defined for each trait. Interviews from all participants were watched in random order and coded by a blinded research assistant. Each trait was assigned one of the codes available, according to the responses given to a fixed subgroup of questionnaire questions and following a strict coding guideline with detailed instructions and sample responses. For example, responses to questions “Draw the planet here”, “Is this how it would look like from a spaceship?”, and “What would it look like?”, were used to code “Earth’s abstract shape” trait (see Supplementary Tables 2 and 3 for a full list of traits and which questions were used to code them). Each trait could be coded in three channels, each based on verbal, drawn and gestural responses, respectively (see Fig. 4a).

The experimental procedure assured that, for each interview, at least 29 out of 44 traits would be coded. These mandatory traits corresponded to aspects explicitly asked in the questionnaire (for instance, the geometrical shape of the earth). The remaining 15 optional traits referred to topics that could be raised spontaneously by children (or not). Thus, they could be waived in interviews where the child may have not expressed knowledge about that particular trait. For example, one of the optional traits, “Round vs flat planet contradiction”, was coded only if the child spontaneously referred to the apparent contradiction between the round earth and the flat ground, but this aspect was not explicitly addressed by any of the questionnaire questions.

### Dimension score and knowledge level

Some traits can be scored from 0 to 1 to quantify knowledge level (see Supplementary Table 3). Coding scheme 2 allowed the description of knowledge with high resolution. However, some knowledge topics, such as the shape of the earth, are represented in several traits, whereas others in one or just a few, like the location of the sea. To prevent different aspects of observational astronomy knowledge from being over- or underrepresented, traits referring to similar topics were combined and their scores averaged, giving rise to 11 dimensions (each of which included at least one mandatory trait). These dimensions were: earth’s location, shape of the earth, shape of the moon, location of other celestial bodies, location of the sky, participant’s location on the earth, depiction and location of the country, antipode’s location, location of the sea, exploration of planet’s surface, and planet’s edge (see Fig. 4a and Supplementary Table 3 for a more detailed description).

Figure 4b shows that average dimension scores show a normal distribution and that it correlates tightly with the average score of Coding scheme 1’s conceptual axes, which are more closely related to previously described mental models of the earth,^26^ *r*(82) = 0.65, *p* = 3.49E−11. A quantitative analysis using the Shapiro–Wilk and Hartigan’s dip tests respectively rejected both normality (*W* = 0.93, *p* = 1.27E−04) and unimodality (*D* = 0.09, *p* = 6.26E−06) for average axis scores, whereas not for average dimension scores (*W* = 0.97, *p* = 0.058, and *D* = 0.03, *p* = 0.840, respectively), which also showed less saturation (Fig. 4b). This indicated that dimension scores provide a measure of conceptual knowledge that is more suitable for assessing knowledge level differences in the sections below.

Score differences between members of a dyad, i.e., between Children M and L (knowledge level *gap*: Δ_ML_), were calculated for each dimension and then averaged:

1 $${\Delta _{ML}^{jk} = \left( {\mathop {\sum}\limits_{i = {\mathrm{1}}}^n {d_i^M} - d_i^L} \right)_{jk}/n},$$

where ${\Delta _{ML}^{jk}}$ is the score difference between Children M and L of the jth dyad in interview k, n is the total number of dimensions (*n* = 11), and ${d_i^M}$and ${d_i^L}$are the ith dimension score for Children M and L, respectively. Score differences between Interviews 1 and 2 of one participant (knowledge level shift: Δ_12_) are calculated analogously:

2 $${\Delta _{{\mathrm{12}}}^j = \left( {\mathop {\sum}\limits_{i = {\mathrm{1}}}^n {d_i^{\mathrm{1}}} - d_i^{\mathrm{2}}} \right)_j/n},$$

where ${d_i^{\mathrm{1}}}$and ${d_i^{\mathrm{2}}}$are the ith dimension score for the jth participant’s Interviews 1 and 2, respectively (see Fig. 1a). In both cases, each dimension score is the average of its three channels’ scores.

### Matching traits and knowledge similarity

To evaluate knowledge similarity, coding of traits was compared between interviews; either between participants or within (between Interviews 1 and 2). Specifically, a trait is considered to match if it was assigned the exact same code in both interviews for at least one of the three channels: verbal, drawn or gestural. We did not consider different codes at the same knowledge level as a match (Fig. 2a).

### Data analysis

Data analysis was conducted using Matlab R2015a for Linux. Two-tailed tests are reported. Shapiro–Wilk test of normality and Hartigan’s dip test of unimodality were run on R 3.0.2.

## Supplementary information

Supplementary Information

## Data Availability

The data sets generated are available from the corresponding authors upon reasonable request.

## Supplementary information

**Supplementary information** accompanies the paper on the *npj Science of Learning* website (10.1038/s41539-019-0051-3).
