# Supplementary material for: Social interaction and conceptual change pave the way away from children’s misconceptions about the Earth
Source: NPJ Sci Learn. 2019 Aug 28;4:12. doi: 10.1038/s41539-019-0051-3 (PMC6713729; doi:10.1038/s41539-019-0051-3)
Supplement: Supplementary file 1 — Supplementary Information [file 41539_2019_51_MOESM1_ESM.pdf]

Supplementary Table 1

Interviews' questionnaire.

Verbal (V) or drawn (D) responses were expected for each question. 30 questions were obligatory (O) and 20 were conditional (C) on previous responses. Original wording in Spanish is shown between parentheses.

Num	Mode	Type	Condition	Question
Q01	V	C	Interview 1 only	Do you have siblings? Older or younger than you? (*¿Tenés hermanos? ¿Mayores, menores?*)
Q02	D	C	Interview 1 only	Write your name with this pen here. (*Escribí tu nombre con esta birome acá*.)
Q03	D	C	Interview 1 only	Very good! Now erase it with the back of the pen. (*¡Muy bien! Ahora borralo, con la parte de atrás de la birome*.)
Q04	V	O		What's the name of our planet? Planet… (*¿Cómo se llama nuestro planeta? Planeta...*)
Q05	V	O		Where do we have to look at to see planet Earth? (*¿A dónde tenemos que mirar para ver el planeta Tierra?*)
Q06	V	O		What shape does the planet Earth have? (*¿Qué forma tiene el planeta Tierra?*)
Q07	V	O		[shape]? How's that? (*¿[forma]? ¿Cómo [forma]?*)
Q08	V	O		But, [shape] like what? Like what thing? (*Pero, ¿[forma] como qué? ¿Como qué cosa?*)
Q09	D	O		Well, draw the planet Earth here. (*A ver, dibujá el planeta Tierra acá.*)
Q10	V	O		Is this how it would look from a spaceship? (*¿Así se vería desde una nave espacial?*)
Q11	D	C	"no" in Q10	What would it look like from a spaceship? (*¿Cómo se vería desde una nave espacial?*)
Q12	D	O		Now, in the drawing you made, where is the Moon? Draw it. (*Ahora, en el dibujo que hiciste, ¿la Luna dónde está? Dibujala.*)
Q13	D	O		And the stars? (*¿Y las estrellas?*)
Q14	V	C	if Moon or stars drawn inside the planet	Then, the Moon/the stars… where are them? (*Entonces, la Luna/las estrellas… ¿dónde están?*)
Q15	V	O		Are there more stars? (*¿Hay más estrellas?*)
Q16	D	C	"yes" in Q16	Where are them? (*¿Dónde hay más estrellas?)*
Q17	D	O		And the Sun? Where is it? Draw it. (*¿Y el Sol? ¿Dónde está? A ver, dibujalo.*)
Q18	D	O		And the sky? (*¿Y el cielo?*)
Q19	D	O		And the clouds? Where are them? (*¿Y las nubes dónde están?*)
Q20	V	C	if Sun, sky or clouds drawn inside the planet	Then, the Sun/the sky/the clouds… where are them? (*Entonces, el Sol/elcielo/las nubes ¿dónde están?*)
Q21	V	O		Are there more clouds? (*¿Y hay más nubes?*)
Q22	D	C	"yes" in Q21	Where are them? (*¿Dónde hay más nubes?*)
Q23	V	O		What was this [pointing at the planet]? (*¿Esto [el planeta Tierra] qué era?*)
Q24	D	O		Very good! Now show me where you are. Draw yourself there. (*¡Muy bien! Ahora mostrame dónde estás vos, y dibujate ahí.*)
Q25	D	C	Q24 not drawn	And where are you in your drawing? (*¿Y en tu dibujo dónde estás?*)
Q26	D	O		Now show me where your house is. Draw it. (*Ahora mostrame dónde está tu casa. Dibujala.*)
Q27	D	O		And a tree? (*¿Y un árbol?*)

Num	Mode	Type	Condition	Question
Q28	V	O		What country are we in? (*¿En qué país estamos?*)
Q29	D	O		Show me where [country name] is. (*Mostrame dónde está el país.*)
Q30	V	O		And you [pointing at self drawing]? Are you in [country name, pointing at it]? (*Y vos ¿estás en el país?*)
Q31	D	C	“no” in Q30	Where are you? (*¿Dónde estás?*)
Q32	D	O		And China? Show me where it is. (*¿Y China? Mostrame dónde está.*)
Q33	D	O		Now draw a person who lives in China. (*Ahora dibujá una persona que viva en China.*)
Q34	V	O		Can that person see the Sun, the Moon and the stars? (*Y esa persona, ¿puede ver el Sol, la Luna y las estrellas?*)
Q35	V	C	“no” in Q34	Why can't she see them? (*¿Por qué no puede verlas?*)
Q36	D	O		And where is the sea? (*¿Y el mar dónde está?*)
Q37	V	C	if sea drawn above objects drawn previously	Are [name objects] beneath the sea? (*¿Estos objetos están abajo del mar?*)
Q38	D	O		Show me where you had drawn yourself. (*Mostrame dónde te habías dibujado.*)
Q39	D	O		Show me where you would get to if you walked straight all day long. What is in there? (*Mostrame dónde llegás si caminás derecho todo el día. ¿Qué hay ahí?*)
Q40	D	C	if planet's perimeter not pointed at yet	Show me where you would get to if you continued to walk straight every day. What is in there? (*Mostrame dónde llegás si seguís caminando derecho todos los días. ¿Qué hay ahí?*)
Q41	D	C	if planet's perimeter not pointed at yet	Show me where is the furthest you can go. What is in there? (*Mostrame dónde es lo más lejos que podés llegar. ¿Qué hay ahí?*)
Q42	V	C	if planet's perimeter not pointed at yet	Can you reach here [pointing at the planet's perimeter]? What is in there? (*¿Se puede llegar hasta acá [punto señalado por entrevistador]? ¿Qué hay ahí?*)
Q43	V	O		Can you continue walking straight from here [planet's perimeter]? (*¿Se puede seguir caminando derecho desde acá?*)
Q44	V	O		Can you fall from here [planet's perimeter]? (*¿Te podés caer desde acá?*)
Q45	D	O		Does the earth end somewhere? Does it have a border? (*¿La Tierra se termina en algún lugar? ¿Tiene un borde?*)
Q46	V	C	“yes” in Q45	Can you reach here [border]? What is in there [border]? (*¿Se puede llegar hasta acá [borde]? ¿Qué hay ahí?*)
Q47	V	C	“yes” in Q45	Can you continue walking straight from here [border]? (*¿Se puede seguir caminando derecho desde acá?*)
Q48	V	C	“yes” in Q45	Can you fall from here [border]? (*¿Te podés caer desde acá?*)
Q49	V	C	if nothing drawn below the planet	What is there here, beneath the earth? (*¿Qué hay acá abajo de la Tierra?*)
Q50	V	C	if nothing drawn above the planet	What is there here, above the earth? (*¿Qué hay acá arriba de la Tierra?*)

Supplementary Table 2

Question mapping into traits.

Mapping of the 50 questionnaire questions (Q) into each of the 44 Coding scheme 2 traits is shown below. Crosses (x) indicate questions whose corresponding responses are used to code the indicated traits. Questions without crosses are not used to code any specific traits. Traits without crosses refer to topics spontaneously raised by children at any moment throughout the questionnaire.

	Trait01	Trait02	Trait03	Trait04	Trait05	Trait06	Trait07	Trait08	Trait09	Trait10	Trait11	Trait12	Trait13	Trait14	Trait15	Trait16	Trait17	Trait18	Trait19	Trait20	Trait21	Trait22	Trait23	Trait24	Trait25	Trait26	Trait27	Trait28	Trait29	Trait30	Trait31	Trait32	Trait33	Trait34	Trait35	Trait36	Trait37	Trait38	Trait39	Trait40	Trait41	Trait42	Trait43	Trait44
**Q01**																																												
**Q02**																																												
**Q03**																																												
**Q04**																																												
**Q05**	X																																											
**Q06**		X	X		X																																							
**Q07**		X	X		X																																							
**Q08**		X	X		X																																							
**Q09**				X	X																	X			X																			
**Q10**				X	X																	X			X																			
**Q11**				X	X																	X			X																			
**Q12**						X	X	X		X			X			X																												
**Q13**									X	X							X																											
**Q14**							X	X	X	X			X			X	X																											
**Q15**									X	X							X																											
**Q16**									X	X							X																											
**Q17**											X	X	X					X																										
**Q18**														X	X	X	X	X		X																								
**Q19**																			X	X																								
**Q20**											X	X	X	X	X	X	X	X	X	X																								
**Q21**																			X	X																								
**Q22**																			X	X																								
**Q23**																																												
**Q24**																					X	X				X																		
**Q25**																					X	X				X																		
**Q26**																							X			X																		
**Q27**																								X		X																		
**Q28**																																												
**Q29**																									X	X	X																	
**Q30**																										X	X																	
**Q31**																										X	X																	
**Q32**																											X																	
**Q33**																												X																
**Q34**																													X	X														
**Q35**																													X	X														
**Q36**																															X													
**Q37**																																											X	
**Q38**																																X	X	X	X	X	X	X	X					
**Q39**																																	X	X	X	X	X	X	X					
**Q40**																																	X	X	X	X	X	X	X					
**Q41**																																	X	X	X	X	X	X	X					
**Q42**																																		X	X	X	X	X	X					
**Q43**																																		X	X	X	X	X	X					
**Q44**																																						X	X					
**Q45**																																		X	X	X	X	X	X					
**Q46**																																		X	X	X	X	X	X					
**Q47**																																		X	X	X	X	X	X					
**Q48**																																						X	X					
**Q49**																																												
**Q50**																																												

Supplementary Table 3

Coding scheme 2 traits.

Responses given to all questions by all participants in Interview 2 were pooled together and used to define the 44 knowledge traits listed below.

Num	Description	Type (1)	Codes (2)	Levels (3)	Scored (4)	Both-coded (5)	Dimension (6)	Theme (7)
Trait01	Where to look to see the Earth	M	4	4	yes	34	earth_loc	Sky
Trait02	Earth's abstract shape	M	4	3	yes	42	earth_shape	Sky
Trait03	Earth's analogue shape	M	4	3	yes	36	earth_shape	Sky
Trait04	Earth's depiction as seen from space	M	3	2	yes	42	earth_shape	Sky
Trait05	Round vs flat planet contradiction	O	2	2	yes	1	earth_shape	Sky
Trait06	Moon's shape	M	4	3	yes	41	moon_shape	Sky
Trait07	Moon's location	M	5	2	yes	41	astro_loc	Sky
Trait08	Moon's multiple locations	O	1	1	no	-	-	-
Trait09	Stars' location	M	8	4	yes	42	astro_loc	Sky
Trait10	Moon/stars and night association	O	1	1	no	-	-	-
Trait11	Sun's location	M	7	4	yes	42	astro_loc	Sky
Trait12	Sun's multiple locations	O	1	1	no	-	-	-
Trait13	Sun's location relative to Moon's	M	4	1	no	-	-	-
Trait14	Sky's depiction	M	4	1	no	-	-	-
Trait15	Sky's location	M	9	4	yes	41	sky	Sky
Trait16	Moon's location relative to sky's	O	2	2	yes	39	sky	Sky
Trait17	Stars' location relative to sky's	O	3	2	yes	41	sky	Sky
Trait18	Sun's location relative to sky's	O	2	2	yes	40	sky	Sky
Trait19	Clouds' location	M	9	5	yes	42	sky	Sky
Trait20	Clouds' location relative to sky's	M	3	1	no	-	-	-
Trait21	Subject's location	M	7	3	yes	41	self_loc	Ground
Trait22	Continental placement of subject	O	2	1	no	-	-	-
Trait23	House's location	M	7	3	yes	32	self_loc	Ground
Trait24	Tree's location	M	7	3	yes	36	self_loc	Ground
Trait25	Country's depiction	M	9	4	yes	42	country	Ground
Trait26	Previous drawings inside country	M	4	3	yes	40	country	Ground
Trait27	China's location relative to Argentina's (*)	M	5	4	yes	42	china	Ground
Trait28	Location of Chinese person	M	7	3	yes	42	self_loc	Ground
Trait29	Sun's, Moon's and stars' visibility from China	M	3	2	yes	37	china	Sky
Trait30	Further comments on sky's visibility	O	5	1	no	-	-	-
Trait31	Sea's location	M	8	5	yes	40	sea	Ground
Trait32	Surface exploration starting point	M	2	2	yes	42	navigation	Ground
Trait33	Spontaneous interaction with planet's perimeter	M	3	2	yes	42	perimeter	Navigation
Trait34	Limits to exploration	M	3	2	yes	42	navigation	Navigation
Trait35	Visibility of the explorable space	M	2	2	yes	42	navigation	Navigation
Trait36	Movements from the planet's perimeter	M	4	3	yes	13	perimeter	Navigation
Trait37	Planet's perimeter as a limit	M	2	2	yes	21	perimeter	Navigation
Trait38	Nature of the perimeter limit	O	6	1	no	-	-	-
Trait39	Planet's finitude	M	5	3	yes	36	perimeter	Navigation
Trait40	Day/night cycle	O	1	1	no	-	-	-
Trait41	Drawing scale limitations	O	1	1	no	-	-	-
Trait42	References to the space hidden behind the planet	O	1	1	no	-	-	-
Trait43	Up and down in the planet's drawing	O	3	1	no	-	-	-
Trait44	Screen frame meaning in drawing	O	2	2	yes	4	navigation	Navigation

(1) Type: Traits which refer to subjects addressed by questionnaire questions were mandatory (M) to code. Traits which refer to topics raised by children spontaneously were optional (O) to code.

(2) Codes: Number of predefined codes. Traits with only one predefined code were either coded or not coded.

(3) Levels: Number of knowledge levels into which codes could be sorted.

(4) Scored: Traits with predefined codes at two or more levels (31 traits in total) were scored: *yes*. Within each trait, more advanced codes were given a higher score. Codes at the same knowledge level were given the same score.

(5) Both-coded: Number of participants for which each trait was coded in both interviews. Only traits coded in both interviews for at least 40 participants were considered for score differences.

(6) Dimension: Scored traits were combined into 11 dimensions: three average channel scores (one per channel) were calculated for each dimension, and these were again averaged to obtain one average score for each dimension.

(7) Theme: Alternatively, scored traits were grouped into 3 themes (see Supplementary Discussion).

(*) China was used because its antipodicty with Argentina (where this study was run) is widely present in culture.

Dimensions: *earth_loc*: earth's location; *earth_shape*: shape of the earth; *moon_loc*: shape of the moon; *astro_loc*: location of celestial bodies; *self_loc*: child's location on the earth; *china_loc*: antipode's location; *navigation*: exploration of planet's surface; *perimeter*: planet's edge.

Supplementary Table 4

Additional analysis to “Changes in children’s knowledge similarity after interaction” section.

Given that, because of the nature of children’s responses, not all traits and channels were coded in all interviews, analysis was repeated to make sure that differences in the number of traits and channels coded in each interview had a negligible effect on the differences in the number of matching traits reported:

Additional Analysis	Condition			Matching dimensions within dyads (between subjects)													
	Relative	No optionals	Both interviews	L1M1		L2M1 (1)				L1M2 (1)				L2M2 (1)			
				M	*SEM*	*M*	*SEM*	*t*(14)	*p*	*M*	*SEM*	*t*(14)	*p*	*M*	*SEM*	*t*(14)	*p*
0 *	No	No	No	10.07	0.72	13.73	0.94	4.91	< .001	10.40	0.74	0.42	.680	13.73	0.89	3.41	.004
1	Yes	No	No	0.28	0.02	0.37	0.03	4.39	.001	0.28	0.02	0.11	.916	0.37	0.03	3.03	.009
2	No	Yes	No	9.80	0.71	13.07	0.86	4.62	< .001	10.20	0.67	0.52	.613	13.20	0.85	3.22	.006
3	No	No	Yes	9.87	0.71	12.80	0.95	4.11	.001	9.93	0.64	0.08	.934	12.67	0.75	3.33	.005

LxMy (e.g. L1M1): traits matching between Child L in Interview x and Child M in Interview y, with x/y: 1 or 2.

Additional analysis conditions:

- Relative: the number of matching traits is relativized to the total number of traits coded (for either one or both members of the dyad).
- No optionals: optional traits (i.e. those not directly addressed by questions in the questionnaire, but rather spontaneously referred to by children) are excluded.
- Both interviews: only channels used in both interviews, for any given child and trait, are considered.

(*) Analysis 0: base analysis, as reported in the main text.

(1) L2M1, L1M2 and L2M2 are compared against L1M1.

Additional analysis to "Response Adoption and knowledge level shifts" section.

Analysis was repeated after dividing the counts of adopted responses by the total number of traits coded, to prevent participants with more coded traits from biasing the results.

One-way ANOVA found significant differences in the number of adopted responses between shift groups (negative, no shift, positive) for Children L, $F(2, 42) = 13.36$, $p = 3.23\text{E}{-05}$. Tukey's HSD test found significantly more adopted responses leading to positive ($M = 0.10$, $SEM = 0.02$) than to either negative ($M = 0.03$, $SEM = 0.01$, $p = 8.42\text{E}{-05}$) or no shifts ($M = 0.04$, $SEM = 0.01$, $p = 3.62\text{E}{-04}$). Again, differences were not significant for Children M, $F(2, 42) = 0.60$, $p = .555$.

Supplementary Figure

Child M knowledge level shift as a function of Child L Interview 1 score

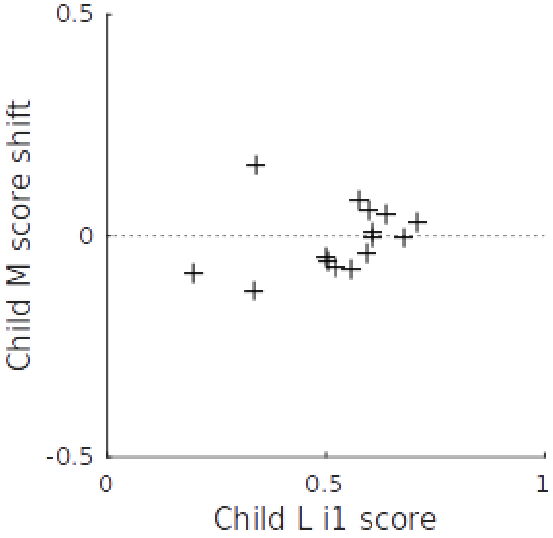

While average Child M knowledge gains were null, a non-significant trend toward knowledge gains was found in Children M interacting with the most knowledgeable Children L, $r(13) = .27$, $p = .331$.

(i1: Interview 1)

Supplementary Discussion

Effect of peer interaction on mental models of the earth

In the main text we showed that the conceptual representation of the earth of children in a dyad progresses, and that this is a consequence of the children with a less advanced representation (Children L) approaching a more advanced one. This result was obtained collapsing conceptual knowledge into 11 dimensions that measure its level.

The questions asked in the interviews probe very different aspects of the conceptual representation of the earth. To investigate if some traits are more prone to change than others, they were alternatively grouped into three themes: *Sky*, *Ground*, and *Navigation*.

Some traits involve our planet and its surroundings: its shape and placement in the universe, and how it relates to other sky (i.e. astronomical and atmospheric) entities, such as the moon, the sun or the clouds. We refer to these traits as belonging to the *Sky* theme. Others relate to the layout of objects, such as buildings, people and continents, on the surface of the earth and relationships among them. We refer to this theme as *Ground*. Finally, a third set of traits refers to how the planet would be revealed to an explorer navigating its surface, what obstacles she/he would find, whether it has an edge, etc. We refer to this theme as *Navigation*. See Supplementary Table 3 for a detailed description of which knowledge traits belong to which theme.

The shift we observed in the Children L's level of knowledge after interaction –progressing towards more advanced representations– was accounted for mostly by changes in *Ground* representations. This was confirmed by independent *t*-tests which showed that Children L's knowledge level shift was found to be significant only in the *Ground* theme, $t(14) = 3.46$, $p = .004$, while not in the *Sky*, $t(14) = 0.69$, $p = .499$, and *Navigation*, $t(14) = 0.00$, $p = 1.00$, themes. No significant shifts were found in any theme for controls or Children M (see Table 1). This also suggests that the lack of change shown in the main text for these two groups was in fact also

observed in each theme and it is not a consequence of compensation of positive and negative changes in different themes.

Table 1: knowledge level shift found for each group of traits or theme

Category	Score shift											
	Control				Child L				Child M			
	M (1E-2)	*SEM* (1E-2)	*t*(11)	*p*	*M* (1E-2)	*SEM* (1E-2)	*t*(14)	*p*	*M* (1E-2)	*SEM* (1E-2)	*t*(14)	*p*
All (1)	0.27	1.65	0.16	.875	7.94	2.47	3.21	.006	-0.86	1.92	-0.45	.662
Sky	4.17	4.66	0.89	.391	2.75	3.96	.69	.499	-4.94	2.60	-1.90	.078
Ground	1.90	2.88	0.66	.523	20.90	6.05	3.46	.004	-2.30	2.02	-1.14	.275
Navigation	-4.17	5.08	-0.82	.429	0.00	5.63	0.00	1.00	-1.11	8.37	-0.13	.896

(1) All: base values as reported in main text, with traits combined into 11 dimensions instead of grouped into themes.

No significant differences in any of the themes were found in Interview 1 scores between peer-interaction groups (Children L and M) and controls, indicating again that the effects we observed could not be accounted for by different baselines before peer interaction/self revision (see Table 2).

Table 2: Interview 1 baseline score in each group of traits or theme

Category	Interview 1 scores										
	Control		Follower		Leader		ANOVA		Tukey's HSD test, *p*-value		
	M	*SEM*	*M*	*SEM*	*M*	*SEM*	F(2, 39)	*p*	Control vs Child L	Control vs Child M	Child L vs Child M
All (1)	0.61	0.03	0.53	0.04	0.72	0.04	10.23	< .001	.169	.053	< .001
Sky	0.73	0.05	0.69	0.03	0.82	0.03	3.53	.039	.730	.220	.034
Ground	0.63	0.06	0.48	0.07	0.81	0.02	10.36	< .001	.124	.070	.001
Navigation	0.38	0.03	0.27	0.05	0.41	0.06	2.28	.116	-	-	-

(1) All: base values as reported in main text, with traits combined into 11 dimensions instead of grouped into themes.

We next wondered what this knowledge level shift in the *Ground* theme meant in terms of the mental models of the earth previously described in the literature (Mali & Howe, 1979; J. Nussbaum, 1979; Sneider & Pulos, 1983; Vosniadou & Brewer, 1992). Multiple regression analysis was used to test if axis scores from Coding scheme 1, which are more closely related to these mental models, significantly predicted the mean *Ground* theme score. Using a least-squares fit, a significant multiple correlation was found, with axis scores explaining 53% of mean *Ground* theme score, R^2 =

.53, $F(4, 80) = 30.1$, $p = 4.11E-13$. Dual/not-dual axis score was found to be a significant predictor of mean *Ground* score ($\beta = 0.45$, $p = 1.61E-12$), while hollow/not-hollow and disc/sphere axis scores were not ($\beta = 0.05$, $p = .365$, and $\beta = 0.09$, $p = .061$, respectively).

Altogether these results suggest that conceptual change from dual to non-dual earth models occurred for Children L after interaction with their more knowledgeable peer. In contrast, we found no evidence to support that this process was promoted for Children M or by controls' simple self revision of the material.
